# Mutagenesis Scanning Uncovers Evolutionary Constraints on Tobacco Etch Potyvirus Membrane-Associated 6K2 Protein

**DOI:** 10.1093/gbe/evz069

**Published:** 2019-03-27

**Authors:** Rubén González, Beilei Wu, Xianghua Li, Fernando Martínez, Santiago F Elena

**Affiliations:** 1 Instituto de Biología Integrativa de Sistemas (I2SysBio), CSIC-Universitat de València, València, Spain; 2 Instituto de Biología Molecular y Celular de Plantas (IBMCP), CSIC-Universitat Politècnica de València, València, Spain; 3 Systems Biology Program, Centre de Regulació Genòmica (CRG), The Barcelona Institute of Science and Technology, PRBB, Barcelona, Spain; 4 The Santa Fe Institute, Santa Fe, New Mexico; 5 Institute of Plant Protection, Chinese Academy of Agricultural Sciences, Beijing, China

**Keywords:** bulk selection, mutagenesis, Potyvirus, TEV, virulence, virus fitness

## Abstract

RNA virus high mutation rate is a double-edged sword. At the one side, most mutations jeopardize proteins functions; at the other side, mutations are needed to fuel adaptation. The relevant question then is the ratio between beneficial and deleterious mutations. To evaluate this ratio, we created a mutant library of the 6K2 gene of tobacco etch potyvirus that contains every possible single-nucleotide substitution. 6K2 protein anchors the virus replication complex to the network of endoplasmic reticulum membranes. The library was inoculated into the natural host *Nicotiana tabacum*, allowing competition among all these mutants and selection of those that are potentially viable. We identified 11 nonsynonymous mutations that remain in the viral population at measurable frequencies and evaluated their fitness. Some had fitness values higher than the wild-type and some were deleterious. The effect of these mutations in the structure, transmembrane properties, and function of 6K2 was evaluated in silico. In parallel, the effect of these mutations in infectivity, virus accumulation, symptoms development, and subcellular localization was evaluated in the natural host. The α-helix H1 in the N-terminal part of 6K2 turned out to be under purifying selection, while most observed mutations affect the link between transmembrane α-helices H2 and H3, fusing them into a longer helix and increasing its rigidity. In general, these changes are associated with higher within-host fitness and development of milder or no symptoms. This finding suggests that in nature selection upon 6K2 may result from a tradeoff between within-host accumulation and severity of symptoms.

## Introduction

### Mutation, Selection, and Mutant Swarms in RNA Virus Populations

RNA viruses exist as complex mutant swarms that result from the combination of three factors: high mutation rates, very short generation times, and very large population sizes (Domingo et al. 2012). These mutant swarms are commonly referred in the virological literature as viral quasispecies (Domingo et al. 2012). Quasispecies theory represent a twist of the classic mutation-selection balance concept from population genetics (Wilke 2005) in which high mutation rates ensure the coupling between genotypes and thus selection operates not on the individual genotype but on the cloud of mutants that are all linked by one or few mutational steps (Bull et al. 2005). The mutant swarm is dominated by a master sequence with the higher fitness surrounded by a cloud of mutants in frequencies that rank according to their fitness. One of the principal tenants of the quasispecies theory is that viral populations replicate close to the so-called error threshold, that is, the highest mutation rate compatible with maintaining genetic information and which is usually proportional to the inverse of the genome length (Bull et al. 2005; Domingo et al. 2012). Increases in mutation rate over the error threshold push the viral populations into a regime known as the error catastrophe in which the frequency of genotypes in the population is not proportional anymore to their fitness, the master sequence disappears and the integrity of genetic information vanishes out (Bull et al. 2005; Domingo et al. 2012). Indeed, this principle provides the theoretical background for antiviral therapies based on lethal mutagenesis (Bull et al. 2007; Perales et al. 2011). The quasispecies population structure is supposed to bestow great adaptive potential to viral populations as the mutant swarm contains genetic variability to respond to environmental fluctuations (Domingo et al. 2012). However, whether such high mutation rates are adaptive per se or a side effect of a parasitic life style that favors fast replication at the cost of low fidelity has been a subject of debate (Elena and Sanjuán 2005; Belshaw et al. 2008). Indeed, the evolutionary fate of mutant swarms and whether they may transit or not to error catastrophe by pushing up mutation rates depends on the proportion of all possible mutations that are lethal, deleterious, neutral, or beneficial (Sardanyés et al. 2014) and on the topography of the underlying fitness landscape (i.e., epistasis; Elena et al. 2010). A considerable amount of effort has been devoted to characterizing the distribution of mutational fitness effects across the genome of RNA viruses by generating collections of random single-nucleotide substitution mutants (e.g., Sanjuán et al. 2004; Carrasco et al. 2007; Visher et al. 2016). However, given the cost of creating such collections, they are limited in size.

To circumvent this limitation and to explore the selective constraints upon an essential viral protein in a high-throughput manner, here we have taken an approach inspired by recent advances in experimental evolution that allow quantitatively tracking the evolutionary dynamics of individual lineages at high resolution (e.g., Levy et al. 2015). Instead of generating mutants by site-directed mutagenesis of a handful of candidate positions, we created in vitro a library of mutants that contains *all* possible single-nucleotide substitutions plus a large number of variants containing more than one mutation of a particular coding gene. The mutant library was then inoculated in a susceptible host and allowed natural selection to do the work of fishing out viable mutants and washing out highly deleterious and lethal ones.

The choice of the viral protein to be studied was based on two principles: 1) It has to play a fundamental role during infection so we expect large fitness effects and 2) it has to be of small enough size so every possible mutation can be created and then the entire coding sequence determined in a single amplicon using Illumina next-generation sequencing, hence linkage among mutations can be directly preserved. We have chosen the 6K2 protein encoded in the genome of *Tobacco etch virus* (TEV; genus *Potyvirus*; family *Potyviridae*; superfamily picorna-like sensu [Koonin et al. 2008]). TEV is a 9.5-kb positive-sense single-stranded RNA virus that encodes for 11 mature peptides, 10 of which are translated as a long polyprotein from a single open reading frame (ORF) and subsequently proteolytically processed by the three viral proteases into the mature peptides (Revers and García 2015). An additional peptide translates from a small ORF that results from a + 2 slippage within the P3 cistron (Chung et al. 2008). The 6K2, which receives its name from its molecular weight of 6 kDa, is a small protein of only 53 amino acids with a transmembrane region encompassing residues about 20–42 (Schaad et al. 1997). There is another protein with similar characteristics, named 6K1. Both proteins are indispensable for viral replication (Merits et al. 2002; Cui and Wang 2016).

### Potyvirus 6K2: An Essential Component of the Virus Replication Complex

Positive-strand RNA viruses replicate on intracellular vesicles that often result from extensive rearrangements of the endoplasmic reticulum (ER) membrane network (Salonen et al. 2005; Miller and Krijnse-Locker 2008; Den Boon and Ahlquist 2010). The role of these membranous vesicles is manifold. First, they provide the scaffold for anchoring the so-called virus replication complex (VRC) to cellular membranes. Second, by restricting the replication process to a small and closed region of the cytoplasm, the concentration of relevant molecules can be high (e.g., replication factors, co-opted host factors, and nucleotides). Third, these vesicles provide a safe environment to protect double-stranded RNA replication intermediates from the action of the DICER endonucleases that trigger the RNAi-mediated cellular antiviral system. Interestingly, some had argued that vesicles containing VRCs composed by viral proteins interacting with a large number of cellular factors, altogether dubbed as *virus replication factories* (Den Boon and Ahlquist 2010), should be considered as the real alive virus (the virion being just a transmission inert stage) (Claverie 2006) and that such virus factories may even be the remote ancestor of the eukaryotic nucleus (Bell 2001; Koonin and Dolja 2013).

6K2 is able to create vesicles by itself even though it needs to interact with other viral and cellular proteins to create functional VRC (Löhmus et al. 2016; Geng et al. 2017). Purifying high-molecular-weight complexes involving 6K2 and using proteomic techniques to identify components, Löhmus et al. (2016) found that 6K2 associates with the potyviral NIb replicase, CI helicase, HC-Pro suppressor of RNA silencing, and the VPg that attaches covalently to the 5′ end of the genomic RNA. In addition, they identified a number of host proteins within these complexes: for example, the isoform 4E of the eukaryotic initiation factor, the poly(A)-binding protein, the eukaryotic elongation factor 1A, the ATP-dependent Clp protease, and the heat-shock cognate 70-1 and 70-3 proteins (Löhmus et al. 2016). Using direct protein–protein interaction techniques (e.g., yeast two-hybrid, coimmunoprecipitation or BiFC), several other host proteins have been identified as 6K2 interactors, including the photosystem II oxygen evolution complex protein NtPsbO1 (Geng et al. 2017), the COPII coatomer component Sec24a protein (Jiang et al. 2015), and the dynamin-related proteins AtDRP2A and AtDRP2B that detach mature clathrin-coated vesicles from plasma membrane (Wu et al. 2018) that are all recruited to the potyvirus VCR.

6K2 anchors the VRC vesicles to the ER membranes (Restrepo-Hartwig and Carrington 1994; Schaad et al. 1997; Cotton et al. 2009) and then migrates to the Golgi apparatus in a COPII-dependent manner (Wei et al. 2010; Agbeci et al. 2013; Jiang et al. 2015). Then, the Golgi-associated 6K2 vesicles move along the actomyosin microfilaments until docking on the outer chloroplast envelopes, wherein they induce membrane invaginations (Cotton et al. 2009; Wei et al. 2010). Cabanillas et al. (2018) have recently shown that impairing the traffic between ER and Golgi by overexpression of the SNARE Sec22 protein resulted in enhanced intracellular virus movement and concluded that turnip mosaic potyvirus (TuMV) replication vesicles bypass the Golgi and take an unconventional pathway for infection that may involve prevacuolar components. Furthermore, 6K2-induced vesicles are involved in cell-to-cell (Grangeon et al. 2013) and long-distance systemic movement of virus-containing vesicles (Rajamäki and Valkonen 1999; Spetz and Valkonen 2004; Wan et al*.* 2015) reminiscent of the egression of animal viruses from cells (Den Boon and Ahlquist 2010). Finally, it has been shown that vesicles are generated from single viral genomes and grow in size as RNA replication and translation continues within the vesicle and viral proteins accumulate in situ (Miller and Krijnse-Locker 2008; Cotton et al. 2009).

Despite the relevance of 6K2 in the infectious cycle of potyviruses, very little is known about the selective constraints operating upon this small protein. In the case of potato virus A (PVA), Rajamäki and Valkonen (1999) have shown that mutation M5V in the N-terminal region of the protein is enough to revert the nonsystemic infection phenotype of isolate PVA-M into a virus capable of spreading systemically. The N-terminal sequence of the 6K2 from TEV contains a conserved diacidic D(X)E motif (Lerich et al. 2011) that is involved in interactions with the COPII coat protein Sec24 in the ER and with the Golgi protein Man1, suggesting a role in trafficking between these two organelles (Hanton et al. 2005). Likewise, mutations in the N-terminal region 6K2 affect the union with Sec24a, blocking export to the ER and thus precluding intracellular and cell-to-cell movement (Jiang et al. 2015). Finally, the 6K2 transmembrane GxxxG motif is key for TuMV infection, and replacement of glycine by valine precludes virus accumulation as a consequence of the relocation of the VRC to the Golgi and the plasma membrane (Cabanillas et al. 2018).

### Short Overview of This Study

The 6K2 mutant library was cloned into an infectious plasmid containing the TEV genome. Then this library was inoculated (by itself or in combination with a fraction of wild-type [WT] TEV genomes) into tobacco plants and infection started. As said above, we expected natural selection to fish out all possible mutations that were viable and not too different in fitness from WT. Nine days postinoculation (dpi), the resulting viral progenies were characterized by Illumina next-generation sequencing and a number of amino acid replacements identified and ranked according to their frequency in the population; in vivo fitness was predicted from the observed changes in frequency. The impact of these mutations in the 6K2 structure and functionality has been evaluated in silico. Finally, we have determined the effect of some of these mutations in TEV infectivity, accumulation, symptomatology, and in 6K2 subcellular location in *Nicotiana tabacum*.

## Materials and Methods

### Synthesis of 6K2 Variants Library

A 206-nt long oligonucleotide was synthesized by TriLink Biotechnologies Inc. (San Diego CA) with the following design: 159 nucleotides (nt) mutated region of 6K2 gene with the 98.8% probability of having WT sequence and 0.4% equal probability of having all three other possible substitutions per position. The immediate upstream 25-nt and downstream 22-nt sequences of mutated regions were synthesized as the WT sequence. The synthesized oligo sequence is 5′-TCACCTGGAAACTATCTATCTCCAAtcagatagcgaagtggctaagcatctgaagcttaaaagtcactggaataaaagccaaatcactagggacatcataatagctttgtctgtgttaattggtggtggatggatgcttgcaacgtacttcaaggacaagttcaatgaaccagtctatttccaaGGGAAGAAGAATCAGAAGCACA-3′, with the uppercase in the sequence indicating the constant flanking regions of the mutated sequence. In between, the lowercase shows the mutated region. Lowercase “a” indicates 98.8% A, 0.4% G, 0.4% C, and 0.4%T; lowercase “g” indicates 98.8% G, 0.4% A, 0.4% C, and 0.4% T; lowercase “c” indicates 98.8% C, 0.4% A, 0.4% G, and 0.4% T; and lowercase “t” indicates 98.8% T, 0.4% A, 0.4% G, and 0.4% C.

### Plasmid Library Preparation

To start with, 1 mg of dry mutagenic oligos were dissolved in 500 μl Milli-Q water as a stock, and further diluted to about 0.5 ng/μl as a working stock. The mutagenic oligo was mixed equimolar with two short DNA fragments overlapping with the mutagenic oligo constant regions for an overlap-extension polymerase chain reaction (PCR). The purpose of this overlap-extension PCR was to introduce *Eco*NI and *Bam*HI restriction enzyme recognition sites for the following ligation step as well as increasing the insert length for the higher efficiency of ligation (supplementary fig. 1*A*, Supplementary Material online). The PCR amplification was performed using Phusion hot-start polymerase (Thermo Scientific, Waltham, MA), first 10 cycles without adding any primers and the next 10 cycles with primers to amplify the full-length products combining all three fragments (supplementary file 1, Supplementary Material online). The PCR product was purified with MinElute PCR Purification kit (Qiagen, Hilden, Germany) after confirming the size of PCR product is correct on a 2% agarose gel. Then, the PCR amplicon was double-digested with *Eco*NI and *Bam*HI-HF enzymes for 1.5 h at 37 °C. Correct size band (449 nt), as an insert for later ligation, was retrieved from agarose gel under the blue light illumination. QIAEX II Gel Extraction kit (Qiagen) was used to purify the DNA from the agarose. The linearized vector was prepared from pMTEV plasmid (Bedoya and Daròs 2010) by cutting and recovering the 10,806-nt fragment from the gel after *Eco*NI and *Bam*HI-HF enzymes double digestion, the same way as the insert preparation. ElectroLigase (New England BioLabs Inc., Ipswich, MA) was used for ligating the two fragments following the manufacturer’s instructions. Ligated products were directly transformed into NEB 10-beta Electrocompetent *Escherichia coli* cells (New England BioLabs Inc.). After recovery in super optimal complete (SOC) for 1 h at 37 °C, 200 μL each SOC media with cells was plated on Luria-Bertani broth (LB) + ampicillin plates. To estimate the total number of transformants, a range of small aliquots of SOC was plated as well on LB + ampicillin plates. We collected an estimate of 0.78–1.2 million transformants from the experiment. Cells were collected from the LB + ampicillin plates after an overnight incubation at 37 °C, with phosphate-buffered saline (PBS) with 1 mM ethylenediaminetetraacetic acid, and plasmids were prepared directly from the cell pellets collected from the overnight plates using Plasmid Midiprep kit (Qiagen). Plasmid concentration was quantified using NanoDrop 2000 (Thermo Scientific).

To verify the quality of the library, 96 colonies were picked randomly for Sanger sequencing after amplifying the mutated region by colony PCR (primers provided in supplementary file 1, Supplementary Material online). Sanger sequencing was performed at UPF Genomics Core Facility (www.upf.edu/web/sct-genomics; last accessed May 4, 2016). The number of mutations and their distributions per colony were examined (supplementary fig. 1*B*, Supplementary Material online).

### Plant Inoculation

The mutant plasmid library was linearized by digestion with *Bgl*II prior to in vitro RNA synthesis using the mMESSAGE mMACHINE SP6 Transcription Kit (Ambion Inc., Austin, TX) following the manufacturer’s instructions to obtain 5′-capped infectious RNA, as described in Carrasco et al. (2007). The third true leaf of 4-week-old *N. tabacum* L. cv Xanthi *NN* plants was mechanically inoculated with 20 μg of transcribed RNA suspended in 500 μL inoculation buffer (50-mM KH_2_PO_4_, pH 7.0, 3% polyethylene glycol 6000, 100 mg/mL carborundum). All symptomatic tissue was collected 9 dpi and stored at −80 °C until analyzed. RNA was extracted from 100-mg homogenized infected tissue using the InviTrap Spin Plant RNA Mini Kit (Stratec Molecular GmbH, Berlin, Germany).

### Illumina Library Preparation

RNA was treated with Turbo DNase and made into first-strand cDNA with SuperScriptIII kit (Thermo Scientific). The primer for this first step is a target-specific primer with an overhang of SP2 Illumina sequence for cluster generation for HiSeq (Illumina Inc., San Diego, CA) (supplementary fig. 1*C*, Supplementary Material online). After treatment with RNaseH, cDNA was amplified with Phusion polymerase (Thermo Scientific) for 25 cycles with Illumina-barcoded primers. In this step, the same forward primer (with an overhang of 6-nt barcode and Illumina P5/SP1) but a different reverse primer (Illumina P7 sequence and sample-specific Illumina barcodes) were used for each sample. Correct size PCR products were collected using the 2% size-select e-gel system, and further desalted with MiniElute PCR Purification kit (Qiagen). Sample libraries were multiplexed into one HiSeq 2500 System (Illumina Inc.) lane at CRG Genomics Core Facility for the 125 pair-end sequencing, for a total read count of 3.1–4.2 millions per sample.

According to the phiX spike-in error rate given by the CRG Genomics Core Facility, the technical error of the HiSeq varies between 0.3% and 1% per site.

### Bioinformatics Pipeline for Analysis of NGS and TEV 6K2 Quasispecies Reconstruction

Read artifact filtering and quality trimming (3′ minimum Q28 and minimum read length of 50 bp) was done using FASTX-Toolkit version 1.01 as implemented in Galaxy (Afgan et al. 2018) and with default parameters. In addition, FASTQ datafiles were also transformed into SAM formatted files using FastqToSam version 2.7.1.0 and BAM-to-SAM version 2.0 as implemented in Galaxy (Afgan et al. 2018) and with default parameters. In any case, reads containing undefined nucleotides (N) were discarded. Two different algorithms were used to reconstruct TEV 6K2 quasispecies. First, QuRe version 0.99971 (Prosperi and Salemi 2012), with default parameters, that uses a heuristic algorithm which matches multinomial distributions of distinct viral variants overlapping across the 6K2 sequence. QuRe incorporates a built-in Poisson error correction method and a postreconstruction probability clustering. The input for QuRe is a FASTA file containing all aligned reads and a reference sequence (also in FASTA format). Second, aBayesQR (Ahn and Vikalo 2018), also with default parameters, that uses a maximum-likelihood framework to infer individual sequences in a mixture via agglomerative hierarchical clustering and has been proved to be particularly efficient detecting low frequency variants. The input for aBayesQR is a SAM file and a reference sequence (in FASTA format). Only variants detected by both methods were retained for subsequent studies. As 6K2 reference sequence to align the reads in both algorithms, we used GenBank accession DQ986288, which corresponds to the TEV-7DA strain used in this experiment.

The fitness value of each selected variant, *W_i_*, relative to the rest of the variants in the quasispecies was evaluated as described in Carrasco et al. (2007). In short, $W_{i}\approx {\left\{\frac{p_{i,t}\left(1-\left[fp_{i,\mathrm{lib},0}+\left(1-f\right)p_{i,\mathrm{wt},0}\right]\right)}{\left(1-p_{i,t}\right)\left[fp_{i,\mathrm{lib},0}+\left(1-f\right)p_{i,\mathrm{wt},0}\right]}\right\}}^{1/t}$, where *p_i_*_,__*t*_ is the frequency of the *i*-variant *t* dpi, *p_i_*_,__lib,__0_ the frequency of the same variant in the mutant library, *p_i_*_,__wt,__0_ the frequency of the variant in the WT population, and *f* the fraction of the library in the inoculated mixture.

### In Silico Structure Predictions and Probability of Membrane Association

Three-dimensional structure predictions were created for WT and mutant 6K2 proteins using the RaptorX server (Källberg et al. 2012; raptorx.uchicago.edu; last accessed May 4, 2019) and then visualized and annotated using tools available at the Jena3D server (Hühne et al. 2007; jena3d.leibniz-fli.de; last accessed May 4, 2019). In addition, transmembrane helices were predicted for the different 6K2 sequences using the methods implemented in the Trans-Membrane Hidden Markov Model (TMHMM) server version 2.0 (Sonnhammer et al. 1998; www.cbs.dtu.dk/services/TMHMM/; last accessed May 4, 2019).

Comparisons between predicted structures for the WT and the mutants 6K2 proteins were performed in the MArkovian TRAnsition of Structure evolution server (Kawabata 2003; strcomp.protein.osaka-u.ac.jp/matras; last accessed May 4, 2019). Two different measures of structural similarity were obtained. *R*_dis_ represents the normalized structural similarity index and ranges between 0% (no overlap in structures) and 100% (complete overlap). dRMS represents the root-mean square deviation (in Å) of distances between Cβ atom positions of aligned residues; the larger the dRMS value, the less overlap among structures (Kawabata and Nishikawa 2000).

The functional effects of the different 6K2 sequence variants detected in this study were explored in silico using the machine learning tools provided in the Screening for Nonacceptable Polymorphisms (SNAP2) web server (Hecht et al. 2015; rostlab.org/services/snap2web/; last accessed May 4, 2019). Using information about the biophysical amino acid properties, sequence, predicted secondary structure, residue flexibility, solvent accessibility, PFAM, PROSITE and SWISS-PROT annotations, predicted binding residues, predicted disordered and low-complexity regions, proximity to N- and C-termini, and statistical contact potentials, SNAP2 provides a score for all possible variants at each residue of the 6K2 protein. The score ranges from −100 in the case of no effect to 100 in the case of maximal effect on the function, regardless it is positive or negative in terms of TEV fitness (Hecht et al. 2015).

### Site-Directed Mutagenesis and In Vitro Transcription

The infectious clone pMTEV contains a full copy of the genome of a TEV-7DA strain isolated from tobacco (GenBank accession DQ986288; Bedoya and Daròs 2010). Eleven mutant genotypes were constructed by site-directed mutagenesis starting from template pMTEV plasmid. Mutagenesis was done using the *Pfu*Turbo DNA polymerase (Stratagene, San Diego, CA), and following the manufacturer’s instructions using the pairs of mutagenic primers listed in supplementary table 1, Supplementary Material online. After Sanger sequencing the mutant genotypes, infectious 5′-capped RNAs were generated in vitro as described above. RNA integrity and quantity were assessed by gel electrophoresis.

### Transient Expression of Mutant 6K2 Proteins in *Nicotiana benthamiana* Leaves and Confocal Laser-Scanning Microscopy

To express 6K2 fused to the yellow fluorescent protein (YFP), the 6K2 cDNA was amplified from pMTEV plasmid with *Pfu*Turbo DNA polymerase (Stratagene) and specific primers including Gateway adapters, and recombined into pDONR207 using BP ClonaseMixII kit (Invitrogen, Carlsbad, CA). After sequencing, 6K2 cDNA was recombined into pEarleyGate101 vector (Invitrogen) using LR ClonaseMixII kit (Invitrogen). The same direct site mutagenesis described above was done to obtain all 6K2 mutant genotypes in this plasmid.

*Agrobacterium tumefaciens* C58 cultures harboring relevant binary constructs were centrifuged and suspended in 10-mM MES pH 5.6, 10-mM MgCl_2_, 150-mM acetosyringone and OD_600_ was adjusted to 1. Transient expression was performed by agroinfiltration into *N. benthamiana* leaves. After 2 days, fluorescence was analyzed in infiltrated leaves using an inverted Zeiss LSM780 inverted confocal microscopy with a CAPO 40×/1.2 objective (Carl Zeiss MicroImaging GmbH, Jena, Germany). YFP-derived fluorescence was monitored by excitation with 488-nm argon laser, and detection windows of 520–550 nm. Imaging processing was performed by ImageJ version 1.8.0_172 (Schneider et al. 2012; imagej.nih.gov/ij; last accessed May 4, 2019).

### Plant Inoculations, Virus Purification, Phenotyping of Infections, and Quantification of Infectious Viral Load

All the inoculations were performed in the virus natural host *N. tabacum* plants. Batches of ten 8-week-old plants were inoculated with ∼5 µg RNA of each viral genotype by abrasion of the third true leaf with 10% carborundum (100 mg/ml). Plants were maintained in a Biosafety Level-2 greenhouse chamber at 25 °C under a 16-h natural sunlight (supplemented with 400 W high-pressure sodium lamps as needed to ensure a minimum light intensity of PAR 50 μmol/m^2^/s) and 8-h dark photoperiod.

After inoculation, plants were visually observed every day for the presence and severity of symptoms and the number of symptomatic plants recorded. A plant was considered as infected if it showed visible symptom of TEV infection. Two different pathogenicity-related traits were estimated from these data. First, the percentage of symptomatic plants at the end of the experiment (i.e., 20 dpi) was used as an estimate of infectivity (*i*). This estimate should be taken as a lower limit of the real *i*, as asymptomatic plants yet infected plants would be missed. However, it has been confirmed in many previous studies that there is a one-to-one association between infection and symptoms development (e.g., Lafforgue et al. 2012), henceforth, we are confident our estimate based on symptoms is not largely deviating from the actual value. Second, infectivity time-series data were submitted to a Kaplan–Meier regression analysis of survival times and the median time to the appearance of symptoms (*ST*_50_) estimated.

Nine dpi virus-infected leaves and apexes from each symptomatic plant were collected and this tissue was frozen with liquid N_2_ and homogenized using a Mixer Mill MM 400 (Retsch GmbH, Haan, Germany). Sap was prepared by adding 1 mL of 50 mM potassium phosphate buffer (pH 7.0) per gram of homogenized tissue. For each sap sample 1:1, 1:5, 1:10, 1:50, 1:100, 1:500, and 1:1,000 serial dilutions were done, inoculating with 10 μl of each dilution four replicates in independent leaves of 4-week-old *Chenopodiumquinoa* Willd plants. Extra care was taken to always inoculate leaves of the same developmental stage. Infectious viral load, measured as the number of lesion-forming units (LFUs) per μL of inoculum, were inferred from the regression of the observed number of local lesions to the dilution factor (Kleczkowski 1950). These experiments were reproduced in two independent blocks in consecutive years (started October 2, 2017 and January 29, 2018, respectively). All mutants were assayed simultaneously in both blocks.

All raw data are available at LabArchives under doi:10.25833/9kqb-e545.

## Results

### Variants Library Characterization and Bulk Selection Experiments

**Fig. 1.**
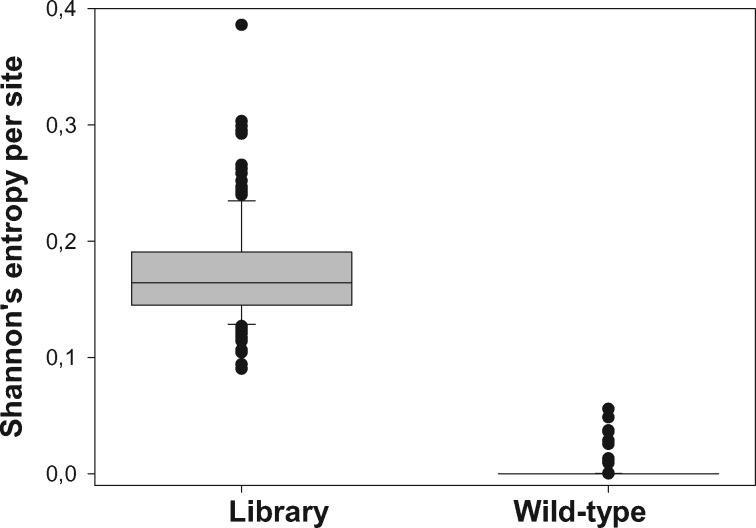
—Average per site variability, measured as Shannon’s entropy, for the variants library and the WT TEV population.

First, we assessed the 6K2 allelic composition present in a TEV population resulting after infecting a *N. tabacum* plant with the WT virus. To do so, we sequenced it using a HiSeq 2500 System (Illumina Inc.). We found 331 different 6K2 variants (haplotypes) out of 2,216,431 valid reads: 98.67% were of the WT sequence, five other variants had a frequency ≥0.10% and two more had a frequency >0.01%. The per site values of Shannon’s entropy (Shannon 1948) ranged between 0 (completely monomorphic site) and 0.0557 with a median value of 0 (fig. 1).

As expected, this genetic composition was in sharp contrast with the composition and variability of the mutant library, wherein we have identified 1,029,895 different genetic variants out of 2,100,648 valid reads obtained. Twenty-five percent of variants contained indels, and the number of point mutations per molecule ranged between 0 (3% of molecules) and 8 (1% of molecules), with a mean of 3.19 (median of 3) and a standard deviation of 1.75 mutations per variant. Individual variants ranged in frequency between 1.62% and 9.71 ×10^−5^%; 42 variants had a frequency ≥0.10%, 112 a frequency ≥0.05%, 797 a frequency ≥0.01%, and all the rest had lower frequencies. In this case, the per site values of Shannon’s entropy ranged between 0.0903 and 0.3860 with a median value of 0.1644 (fig. 1), being the difference with the WT variability highly significant (Wilcoxon signed rank test *V *=* *12246, *P *<* *0.0001).

When the library was inoculated in 25 tobacco plants, none resulted in symptoms, whereas all 10 plants inoculated with the WT virus developed the normal symptoms associated with TEV infection 9 dpi. Given the tremendous genetic diversity in the library, this lack of infectivity supports the concept of the quasispecies error catastrophe (Domingo et al. 2012) and suggests that the frequency of the WT 6K2 sequence, plus any other viable sequences, was too low in the mutant library to establish infection at all or to sustain population growth and hence the virus extinguished as a result of entering into the error catastrophe regime (Bull et al. 2007; Domingo et al. 2012).

In an attempt to avoid this error catastrophe effect, we generated mixtures of the library and RNA transcribed from the WT infectious clone pMTEV with increasing ratios of the library: 50%, 75%, and 95%. Inoculation with all three mixtures resulted in symptomatic infections (ten plants each). After purifying the resulting viral populations 9 dpi and deep sequencing them as above, the number of valid reads were 3,342,356, 3,840,036 and 2,907,325, respectively. In the three populations, the frequency of the WT 6K2 were very similar: 90.4%, 90.6%, and 90.5%, respectively, and significantly lower than observed in the WT population (see previous paragraph), meaning that a number of alternative variants coexisted in these mixed populations at noticeable frequencies. Using two different quasispecies-reconstruction algorithms (QuRe and aBayesQR; see the Materials and Methods section for details), we identified 23 variants with frequencies in the range 0.32–0.01% depending on the particular inoculation experiment (table 1). Some of the mutations (though not necessarily the variants) were recurrently observed in different inoculated plants (e.g., nonsynonymous mutations U95G, G101A, G104U, and C132A and synonymous mutation C157A). Another remarkable observation is that 18 of these 23 viable mutant alleles affect the stretch of amino acid residues from 32 to 39, all belonging to the predicted transmembrane domain of the 6K2 protein.

**Table 1:** Twenty-Three 6K2 Variants Identified by Illumina Sequencing after Infection

Amino Acid Substitution	Nucleotide Substitution	Protein Localization	Type of Amino Acid Substitution	Percentage of Library in Inocula	Relative Fitness
*I32S*	U95G	Transmembrane	Nonpolar to polar	95	0.5122
I32S	U95A	Transmembrane	Nonpolar to polar	75	0.5837
*I32S/G33D*	U95G/G98A	Transmembrane	Nonpolar to polar/nonpolar to acid	50	1.5669
I32T	U95C	Transmembrane	Nonpolar to polar	50	0.8056
				95	0.5495
Synonymous	U99G			50	0.9726
Synonymous	U99G/U102G			95	∞^a^
Synonymous/synonymous/D44E/F46I	U99G/U102G/C132A/U136A	C-terminal	Conservative acid/conservative hydrophobic	95	∞^a^
G34C	G100U	Transmembrane	Nonpolar to polar	50	0.8869
*G34D*	G101A	Transmembrane	Nonpolar to acid	95	1.1500
*G34V*	G101U	Transmembrane	Conservative nonpolar	50	0.7607
*G34R/G35A*	G101C/G104C	Transmembrane	Nonpolar to basic/conservative nonpolar	95	1.3716
*G34S/A39S*	G101A/G116U	Transmembrane	Nonpolar to polar/conservative polar	50	1.8255
*G35V*	G104U	Transmembrane	Conservative nonpolar	75	0.5239
				95	0.5319
W36C	G108U	Transmembrane	Hydrophobic to polar	95	0.5194
*A39E*	C116A	Transmembrane	Nonpolar to acid	95	0.4547
*A39V*	C116U	Transmembrane	Conservative nonpolar	95	0.4897
*D44E*	C132A	C-terminal	Conservative acid	50	0.8467
*D44E/F46L*	C132A/C138A	C-terminal	Conservative acid/hydrophobic to nonpolar	95	1.2397
Synonymous	A147U			50	0.7986
Synonymous	C157A			75	0.5053

Note.—Italic entries indicate 11 haplotypes constructed by site-directed mutagenesis for further experiments.

a Infinity results from an apparent zero frequency of the haplotype in the inoculation mix.

Using the observed change in frequency of all 23 variants between the inoculation mixture and the viral population recovered 9 dpi, it was possible to calculate their relative fitness values in the system (see the Materials and Methods section for details). Estimated fitness values were in the range 0.455–1.826 (median = 0.780), with a mean value of 0.845 ± 0.398 (table 1). Five variants have relative fitness higher than WT: G34S/A39S, I32S/G33D, G34R/G35A, G34D, and D44E/F46L (table 1). Four variants only contain synonymous changes. Eleven of the variants containing nonsynonymous changes, including the five with beneficial fitness effects, were introduced by site-directed mutagenesis into the WT infectious clone pMTEV and their biological properties further characterized.

### Effect of Mutations on Predicted Protein Structure and Functionality

**Fig. 2.**
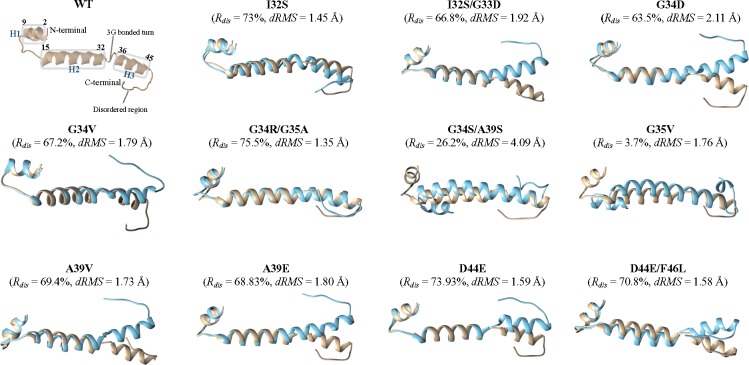
—Comparison of the predicted ternary structures for the WT (in gold) and the mutant (in blue) 6K2 peptides. As measures of structural similarity, the *R*_dis_ and dRMS values are reported. The upper most left panel shows the predicted structure of the WT 6K2 sequence; the three α-helices and other relevant motifs are marked with boxes.

The ternary structure of the WT and mutant 6K2 proteins was evaluated using the RaptorX server (Källberg et al. 2012) and structural similarities evaluated using the MArkovian TRAnsition of Structure evolution server (Kawabata 2003). Figure 2 shows the results of these studies. The predicted folding of WT 6K2 is characterized by the existence of three α-helices, H1 (residues 2–9), H2 (residues 15–32), and H3 (residues 36–45); H1 and H2 are separated by a stretch of five amino acids with low structural complexity (residues 10–14) and H2 and H3 are separated by a bonded turn of three glycine (residues 33–35). The C-terminal part of H1 ends with amino acids K10 involved in a hydrogen bonded turn and L11 bending out. The C-terminal part of H3 ends with F46 also bending out. Residues 46–53 are a disordered region that may confer flexibility to the C-terminal region of the protein. Besides some minor details that affect the C-terminal parts of H1 and H3, the 11 mutants can be classified into two categories according to the separation between H2 and H3: Those that preserve the three α-helices and those that fuse H2 and H3 into a long helix. Mutants A39V, D44E, and D44E/F46L belong to the first category, whereas all others belong to the second (fig. 2). Structural similarities were quantified using the *R*_dis_ and dRMS scores (Kawabata and Nishikawa 2000). A large overlap corresponds to *R*_dis_ = 100% and dRMS = 0, decreasing structural overlaps translate into smaller *R*_dis_ and larger dRMS values. G35V shows an inconsistency among the two values, having the smallest *R*_dis_ but not the largest dRMS values observed. Besides this particular case, the most dissimilar fold corresponded to the double mutant G34S/A39S and the most similar one to the double mutant G34R/G35A (fig. 2).

The TMHMM algorithm (Krogh et al. 2001) predicts that the WT 6K2 has a transmembrane domain encompassing amino acids 20–42. This transmembrane domain coincides with H2 and H3 predicted above and leaves H1 outside membranes. Nine of the 11 viable genotypes we examined contain mutations located in this transmembrane domain. TMHMM also shows that all 11 variants had WT-like predicted transmembrane domains, thus confirming that the changes in folding discussed above do not affect the trans-membrane properties of the protein. Therefore, we reason that this domain is essential for the activity of the protein and thus a change in its conformation may have a strong impact in its functioning.

**Fig. 3.**
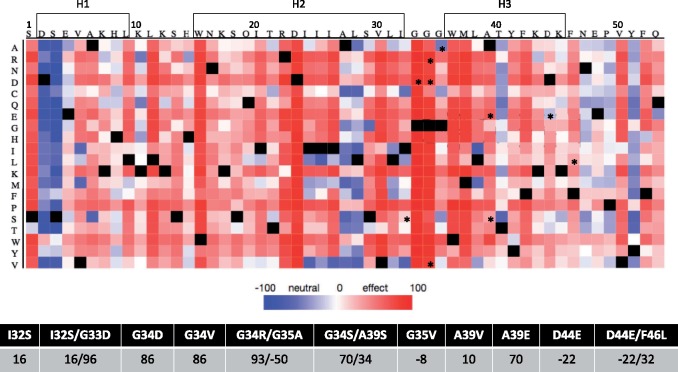
—In silico evaluation of the potential functional effect of every possible amino acid replacement on each residue of 6K2. Columns represent the 6K2 residues (indicated in the top) and rows the possible changes. Hot colors (red) represent strong functional effects, whereas cold colors (blue) represent neutral changes. Black squares represent no amino acid change. Mutations studies in this work are indicated with asterisks. The table below indicates the scores for each of the 11 6K2 mutants studied. Amino acids involved in α-helices H1, H2, and H3 are indicated with boxes.

Next, we sought to evaluate in silico the expected effect of the observed mutations in the 6K2 function. To this end, we used the neural network-based classifier implemented in the SNAP2 webserver (Hecht et al. 2015). The aim of this computational approach is not to address the question of whether or not an allele improves the fitness of TEV but just if it has a possible impact on 6K2 function. Figure 3 shows the results of this study. Interestingly, any amino acid replacement affecting residues W15 and D23 (H2), G33 and G34 (bond between H2 and H3), and W36 (H3) is predicted to have a strong effects on 6K2 function. By contrast, positions D2 and S3 (N-terminal part of H1), T40 (center of H3), and Y51 (disordered C-terminal region) are those with more tolerance to changes.

### Subcellular Localization of 6K2 Mutants

**Fig. 4.**
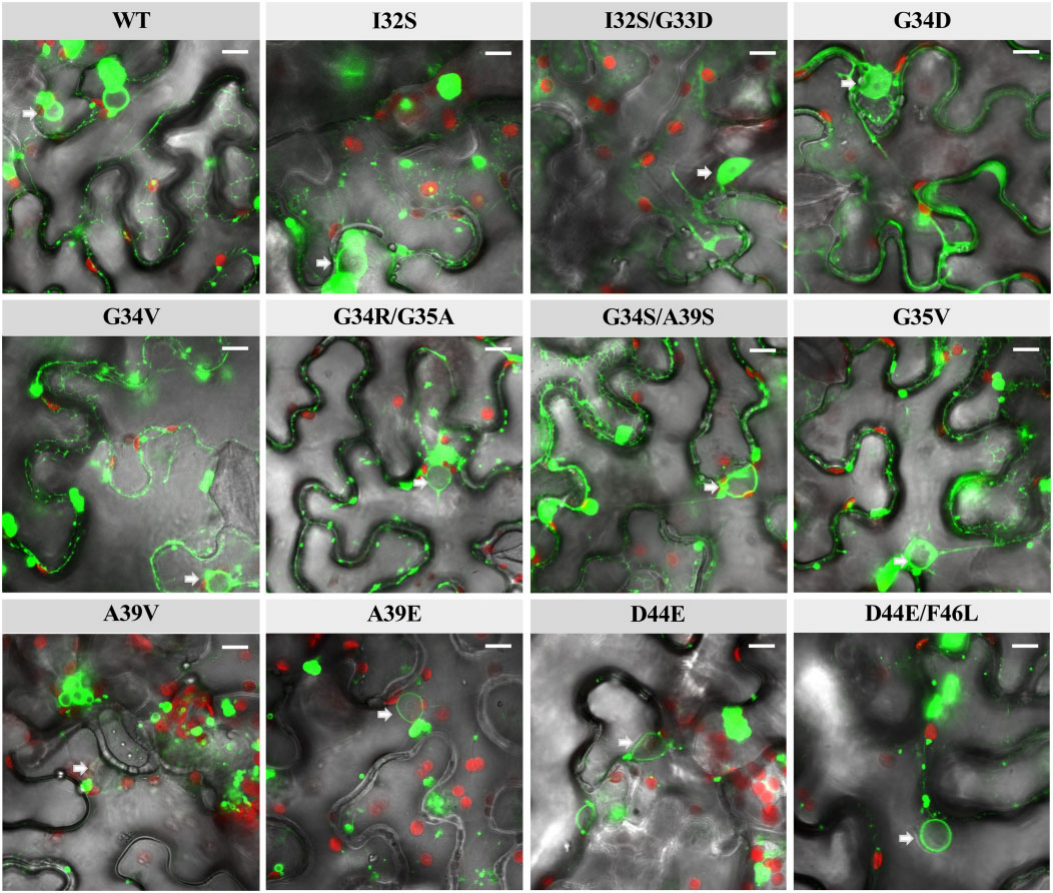
—Confocal microscopy imaging of *Nicotiana benthamiana* leaf epidermal cells expressing 6K2::YFP. Each image panel shows the localization of the WT protein and its variants (1–13). The position of nuclei is indicated with an arrow. Red objects are chloroplasts. White scale bar represents 10 μm.

As mentioned in the Introduction, 6K2 has the ability to induce vesicles by itself and these vesicles form VRC by associating with other viral host proteins. As we have illustrated in the previous section, 6K2 is predicted to be hydrophobic and transmembrane associated and hence the vesicles it creates are localized in the ER membranes and around the nucleus and organelles (Cotton et al. 2009; Wei et al. 2010). We have engineered C-terminus YFP-tagged versions of WT and the 11 mutant proteins to explore, using confocal microscopy, whether mutations have an effect on the subcellular localization of the proteins. 6K2-YFP was transiently expressed by agroinfiltration (see Materials and Methods). Figure 4 shows representative images for all the mutant genotypes. We found that nine of the mutants have the same subcellular localizations as the WT protein, localizing in perinuclear ER membranes (nuclei are pointed with arrows) and generating vesicles of cytoplasmic localization. By contrast, mutants I32S/G33D and G34D show a different intracellular distribution: In addition to distribute homogeneously along the periplasmic membrane, they are also highly concentrated in the nucleoplasm (fig. 4). Interestingly, these two genotypes have a predicted fused H2 and H3, whereas inducing a change in the angle of the region corresponding the H3 and the C-terminus of the protein respect to the WT configuration (fig. 2). In concordance, these two genotypes also obtained large scores of functional changes in the SNAP2 analyses.

As an interesting corollary of these experiments, all mutations rescued from the mutant library are able to form vesicles and anchor to membranes, thus suggesting that strong selection for these two characteristics is at play.

### Phenotypic Properties of 6K2 Mutants

**Fig. 5.**
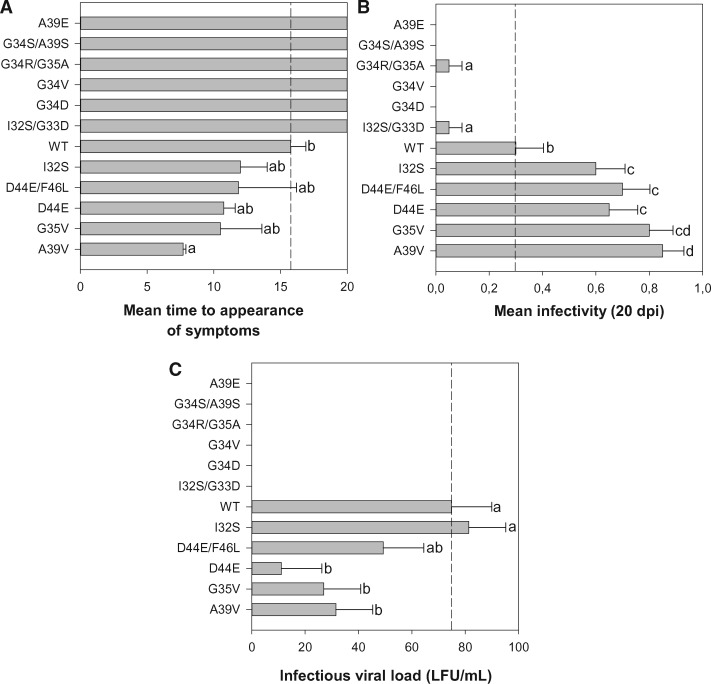
—Phenotypic characterization of the different TEV 6K2 mutants. (*A*) Mean time to symptoms development estimated from the Kaplan–Meier regression. For those genotypes that did not showed symptoms at the end of the experiment, 20 dpi represents the lower bound of the estimated mean time (upper bound being +∞). (*B*) Mean infectivity 20 dpi (*n *=* *10 plants inoculated). (*C*) Infectious viral load estimated by means of *Chenopodium quinoa* local-lesion assay method. In all cases, the dashed vertical line corresponds to the mean phenotypic value of the WT TEV. Mutants are ordered in the ordinate axis to better illustrate the statistically homogeneous groups.

In order to phenotype the 11 genetic variants of 6K2 studied in the previous sections in the natural host *N. tabacum*, two independent infectivity assays were performed using RNA transcripts generated in vitro as inocula. Five of the 6K2 variants (I32S, G35V, A39V, D44E, and D44E/F46L) were infectious in both assays. However, these variants showed differences among them in terms of symptomatology and progression of infection. As can be seen in supplementary figure 2, Supplementary Material online, the TEV carrying different 6K2 mutants induced symptoms that were distinguishable from those induced by the WT virus, all inducing symptoms that were equal or milder and that were visible at earlier or later dpi than those characteristic of the WT TEV. Besides these differences in the symptomatology, the 6K2 mutants also induced visible symptoms at different dpi (fig. 5*A*). A Kaplan–Meier survival analyses was performed to evaluate differences in the dynamics and timing of symptom development. This analysis showed that genotype A39V induced symptoms faster than the WT: on average, a plant infected with the A39V mutant developed visible symptoms ∼8 dpi compared with the average of ∼16 dpi necessary for the WT (fig. 5*A*). The dynamics of symptoms development for mutants I32S, G35V, D44E, and D44E/F46L were equivalent to that observed for WT (fig. 5*A*; post hoc Bonferroni test *P *>* *0.05). The rest of mutants developed symptoms much slower than WT or had not developed symptoms at all 20 dpi when the experiment was concluded (fig. 5*A*). A generalized linear model (GLM) was fitted to the mean time to the appearance of symptoms data. The model used genotypes as a random factor, experimental blocks as replicates, and a Normal probability distribution with an identity link function. Highly significant differences in the time to symptoms appearance among genotypes were observed (*χ*^2^ = 156.597, 11 d.f., *P *<* *0.001). A post hoc sequential Bonferroni test classified the genotypes into three groups (fig. 5*A*).

As a second phenotypic trait, we evaluated the infectivity, that is, the proportion of infected plants 20 dpi. A GLM with genotypes as random factor, experimental blocks as replicates, and a binomial probability distribution with a logit link function found highly significant differences in infectivity among genotypes (fig. 5*B*;*χ*^2^ = 151.034, 11 d.f., *P *<* *0.001). Genotypes G34D, G34V, and A39E did not induce any symptom 20 dpi and thus we conclude that they were not infectious. Genotypes I32S/G33D and G34R/G35A show a low infectivity (10%) compared with WT. A post hoc sequential Bonferroni test classified the rest of genotypes into three categories, with five of the mutants being significantly more infectious than WT, with the A39V variant being the most infectious (85%, almost three times more infectious than WT). Genotypes I32S, D44E, and D44E/F46L had intermediate infectivity values between WT and A39V variant. The infectivity results negatively correlate with the time required to develop symptoms (Pearson’s *r* = −0.991, 10 d.f., *P *<* *0.001): suggesting that those genotypes inducing symptoms faster were also more infectious (e.g., A39V).

Finally, the third phenotypic trait associated with infection that we characterized was the infectious viral load, measured as the number of LFUs per given amount of infected tissue in a local-lesions assay in fully expanded leaves of *C.quinoa*. LFUs were inferred from the regression of the observed number of local lesions to the dilution factor (Kleczkowski 1950). For six genotypes (fig. 5*C*), we found no local lesions produced in the *C. quinoa* leaves and thus concluded that the amount of infectious viral particles produced in the infected source tobacco plants was null or below the detection limit of the local-lesion technique. Among those genotypes for which lesions were found, *N. tabacum* plants inoculated with the WT produced, on average, 76.81 LFU/ml, whereas mutant D44E shows the lowest value (11.09 LFU/ml) and mutant I32S the highest one (81.29 LFU/ml) (fig. 5*C*). A GLM with genotypes as random factor, experimental blocks as replicates, and a Normal probability distribution with an identity link function revealed highly significant differences in infectious viral load among 6K2 mutant genotypes (*χ*^2^ = 25.665, 11 d.f., *P *=* *0.007). A post hoc sequential Bonferroni test classified the viable genotypes into two categories, those that accumulate similar amount of infectious viral particles than WT (I32S and D44E/F46L) and those that accumulate significantly less (G35V, D44E, and A39V). Comparing the three panels in figure 5, we see that infectious viral load was weakly though significantly correlated with the other two phenotypic traits analyzed. First, it was negatively correlated to the mean time to symptoms development (Pearson’s *r* = −0.582, 10 d.f., *P *=* *0.047), meaning that the more infectious viral particles accumulated, the faster the symptoms might appear. Second, infectious viral load was positively correlated to infectivity (Pearson’s *r *=* *0.584, 10 d.f., *P *=* *0.046), meaning that more infectious genotypes might also result in a larger accumulation of viral particles.

### Integrating Structural Changes with Phenotypic Effects

Figure 5 shows two groups of genotypes: those inducing symptomatic infections and those resulting in asymptomatic or no infection. Indeed, using “presence of symptoms” as a binary factor, we can ask whether differences exist between these two classes in terms of SNAP2 scores (in the case of the double mutants, assuming dominance and using as score the maximum value of the pair) and in vivo replicative fitness. For the case of SNAP2 scores, the average score for the genotypes resulting in asymptomatic or no infections was 86.20 ± 4.5 (±1 SEM), whereas it was 14.0 ± 11.4 for those inducing symptomatic infections, the difference highly significant (2-samples *t*-test with equal variances: *t*_10_ = 5.093, *P *<* *0.001), thus confirming that larger functional changes of 6K2 are associated with milder or no infection, whereas small effect or neutral changes are phenotypically closer to the WT infection. For the case of competitive fitness values, the results are equivalent. The average fitness value for genotypes not inducing a symptomatic infection (or more likely being not infectious) was 1.335 ± 0.182, whereas the value for symptomatic infections was 0.726 ± 0.117, being the difference also significant (*t*_10_ = 2.959, *P *=* *0.014).

**Fig. 6.**
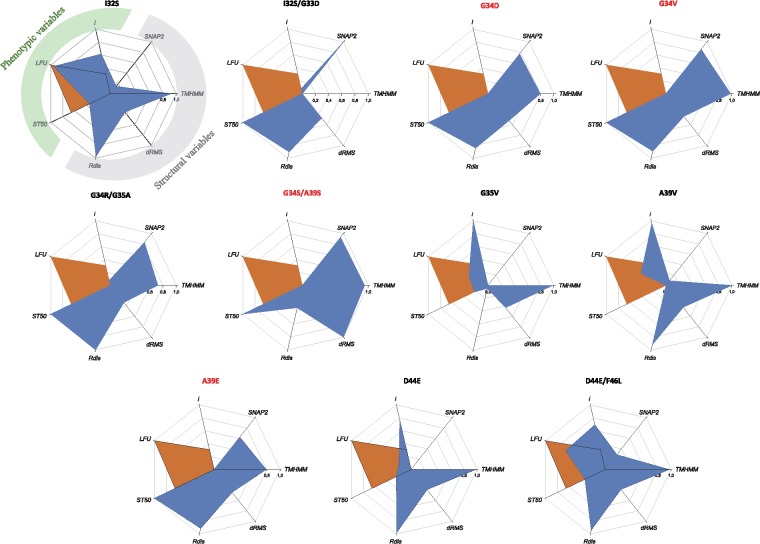
—Radar plot summarizing all the structural and phenotypic variables estimated for each 6K2 mutant. Trait values have been normalized to a zero to one scale. In all plots, the WT values are included in brown as reference. The three phenotypic traits (infectivity *i*, viral load LFU, and mean time to symptoms ST_50_) are placed at the left side of the heptagon (highlighted in green in the first panel). For those genotypes that did not showed symptoms at the end of the experiment, ST_50_ → +∞. The four structural traits (SNAP2, transmembrane score TMHMM, dRMS, and the normalized structural similarity index *R*_dis_) are placed at the right side of the heptagon (highlighted in gray in the first panel). Nonviable mutants are indicated in red.

Finally, to better integrate the four structural variables and the three phenotypic traits, we generated the radar plots shown in figure 6. The four structural variables are placed in the right side of the heptagon (highlighted in gray in the first panel) and the three phenotypic ones in the left side (highlighted in green). In all panels, the combination of WT normalized variables has been indicated by the brown area. Six similar patterns can be readily identified: 1) mutants G34D, G34V, G34R/G35A, and A39E all show the strongest structural perturbations (*R*_dis_ and dRMS) and functional effects (SNAP2), which correlate with very low or no infectivity (*i*), undetectable viral loads (LFU) and the longest times to symptoms development (*ST*_50_; if any at the end of the experiments). Indeed, all but one (G34R/G35A) have been classified as nonviable. 2) I32S and D44E/F46L with strong effects on transmembrane properties (TMHMM), large structural distances with WT, yet small functional effects that translate into moderate infectivity, high viral load and short times to symptoms development. 3) A39V and D44E show strong effects on transmembrane properties and structural differences with WT that are not translated into predicted functional effects yet had no negative effect in infectivity, significantly reduce the time to symptoms development and have a strong negative effect on accumulation. 4) I32S/G33D mutant has no effect on the transmembrane properties but an overall effect on folding similarity with WT that translates into a strong predicted functional effect. This effect is seen in terms of no infectivity, undetectable viral loads and no symptoms development. 5) Mutant G34S/A39S does not differ much from WT folding though it has a stronger transmembrane prediction and large functional effects which result is low infectivity and accumulation and long times to symptoms to be developed. Finally, 6) mutant G35V shows the most distinctive pattern: a strong effect on transmembrane properties which has no predicted functional effect though it increases infectivity, fastens the developments of symptoms and produce moderate accumulations (lower than WT).

## Discussion

Potyvirus 6K2 protein is an essential component for the formation of VRC and hence a key element to successfully complete the infection cycle (Restrepo-Hartwig and Carrington 1994; Rajamäki and Valkonen 1999). Consequently, it has attracted a wealth of attention during recent years. These studies have mainly focused on disentangling the mechanisms by which 6K2 forms vesicles by modifying the structure of ER and other endomembrane complexes (Agbeci et al. 2013; Jiang et al. 2015), how these vesicles move along the cytoskeleton microfilaments (Cotton et al. 2009) to neighboring cells (Grangeon et al. 2013) and long distance (Spetz and Valkonen 2004; Wan et al. 2015), and identifying viral and cellular components that are required for 6K2 function (Löhmus et al. 2016; Geng et al. 2017; Movahed et al. 2017). However, no attention has been paid to the evolutionary constrains that should be operating upon this essential small protein. To tackle this question for the first time, we have taken an experimental evolution approach. We began our work with the generation of a library of TEV mutants carrying almost every possible mutation in 6K2. Then, we inoculated the mutant virus’ library into a plant host and allowed all variants to compete with each other and with different proportions of the WT. It should be expected that natural selection would fish out those variants that are viable and, in the absence of other selective forces such as random drift or spatial stratification, rank their population abundance according to fitness. Finally, using high-throughput Illumina sequencing, we characterized the composition of the evolved populations and estimated the fitness values of each surviving 6K2 haplotype. By doing so, we have identified 23 different variants some of which were as fit as the WT TEV, some beneficial and some slightly deleterious. Some of the mutations pervasively appearing in different experiments (e.g., I32T, I32S, D44E, and G35V) or different mutations affecting the same amino acids sites (e.g., I32, G34, G35, A39, and F46). Out of these, and based on their fitness estimates, we selected 11 6K2 mutants for further biological characterization.

As mentioned, some amino acid residues were more tolerant to mutations than others. Interestingly, most of the identified amino acid replacements were located in the transmembrane domain of 6K2 that encompasses residues 20–42 (Schaad et al. 1997). Out of 17 unique amino acid substitutions (table 1), none was located in the N-terminal domain that contains α-helix H1, 14 were located in the transmembrane domain that contains α-helices H2 and H3 and three affected the C-terminal disordered domain. This distribution of amino acid replacements per domain significantly deviates from what should be expected by chance (Fisher’s exact test, *P *<* *0.001), showing an enrichment in mutations in the transmembrane domain and a depletion of mutations in the two extra-membrane domains, most significantly in the N-terminal one. This provides evidences for purifying selection upon the N-terminal region being stronger than in the other protein domains. This hypothesis is further backed up by previous observations by Rajamäki and Valkonen (1999) and Spetz and Valkonen (2004), indicating that mutations in the N-terminal domain strongly affect the ability of PVA to move systemically, and by Jiang et al. (2015) that found the N-terminus domain of 6K2 essential for ER export and vesicle formation of TuMV.

It is remarkable that several of the mutations found affected the run of three glycines (residues 33–35) involved in the bonded turn that separates H2 and H3. These mutations induced a conformational change resulting in the fusion of H2 and H3 into a longer α-helix, which may increase the rigidity of the transmembrane domain. In addition, this conformational change may contribute to better solubilization of 6K2, allowing entry to the nucleus and reducing to some extent their ability to become integral membrane proteins. Other mutants with large SNAP2 scores (e.g., G34R/G35A and G34V; fig. 3) by contrast, do not suffer the angle torsion affecting H3 and C-terminus. Furthermore, mutations G33D and G34R are the two predicted to have the largest functional effect by the SNAP2 classifier (fig. 3), they both replace the small nonpolar radical of glycine with a large charged radical (negative and positive, respectively). They both appeared linked to other mutations I32S/G33D and G34R/G35A that also affect the bonded turn. I32S contributed an additional polar radical and G35A retains a small nonpolar radical. These mutations affecting the rigidity of the transmembrane α-helix have a negative impact in the dynamics of virus accumulation and symptoms development (fig. 5), yet surprisingly I32S/G33D and G34R/G35A mutants have some of the largest beneficial fitness effects measured in vivo from the change in frequency data (table 1). Supporting the importance of this glycine-rich motif, Cabanillas et al. (2018) have recently shown for TuMV that mutations in this motif cause 6K2 to accumulate in the Golgi apparatus and plasma membrane. Indeed, glycine by valine mutants accumulate in the apoplastic side of the plasma membrane, in contrast to the WT protein that accumulates in the cytoplasmic side.

Mutations A39V and G35V showed the most virulent phenotypes (more infectious and shorter timing to symptom development), though they accumulated less infectious units than WT (fig. 5) and had estimated fitness values approximately half of the WT (table 1). A39V induces a torsion in the molecule that affects the region from H3 to the C-terminus (fig. 2), although this apparently major structural change is associated with a relatively small functional effect according to SNAP2 (fig. 3). Likewise, mutation G35V induces a torsion that affects the orientation of the H1 at the N-terminal part of the molecule and fuses H2 and H3; this structural change was scored as relatively neutral (fig. 3) by SNAP2.

At a first glimpse, the results obtained for predicted functional effects explain quite well the observed disease phenotypes but are at odds with the estimated fitness values. However, three factors should be taken in consideration to draw a complete picture: 1) SNAP2 estimates refer to potential functional changes in the protein itself out of its biological contexts (i.e., interactions with other viral and host factors). 2) Our estimates of within-host fitness from the Illumina data are based on changes in frequency from the inoculum to the sampling time. We cannot rule out that some of mutations in 6K2 rose in frequency by reasons other than an inherent beneficial fitness effect (e.g., drift or a selective hitchhiking). 3) The fitness estimates were obtained in the context of a complex quasispecies in which the master 6K2 sequence corresponded to the WT one. The effect of these mutations thus is modulated by and depends on the composition of the cloud of mutants. By contrast, the virulence assays were done with different quasispecies compositions resulting from the replication of the corresponding infectious clone and thus the master sequences correspond to the mutant sequence.

### Some Considerations about the Bulk Selection Experiments and Fishing Out Potentially Beneficial Alleles

Two aspects of these experiments are worth discussing. First, the library was not infectious by itself. This is not an unexpected result given the dynamic properties of RNA virus mutant swarms presented in the Introduction. Quasispecies theory predicts the existence of an error threshold beyond which the fitness class distribution disappears into a new state, the error catastrophe, in which the frequency of mutants is not determined by their fitness (Bull et al. 2005; Domingo et al. 2012). Increasing mutation rate pushes the viral population across the error threshold. A viral population replicating in the error catastrophe range is doomed to extinction in a process dubbed as lethal mutagenesis in the quasispecies literature and as mutational meltdown in the classic population genetics literature (Bull et al. 2007). Here, by generating all possible single-nucleotide substitution mutants (plus some with higher number of mutations) we have artificially created a population that is already into error catastrophe: the diversity composition in the library is far larger than observed for the WT quasispecies. To rescue the library from error catastrophe and establish a symptomatic infection, it was sufficient to mix it with as low as 5% of WT TEV.

Second, we have detected a limited number of potentially viable genotypes (23) (table 1), five of which have fitness values estimated to be larger than the fitness of WT, whereas others have about the same fitness (e.g., I32T or G34C) and some clearly much lower fitness (e.g., G35V or W436C). Finding beneficial mutations at a frequency high enough as to be detected by the Illumina technique is not surprising. What is more surprising is the finding of largely deleterious 6K2 alleles (e.g., A39E, G34S/A39S, and G34D) at noticeable frequencies. Five possible, nonmutually exclusive explanations, can be brought forward: 1) These deleterious mutations are linked to a beneficial one elsewhere in the genome that is being positively selected and are hitchhiking despite their negative effect. 2) The deleterious effect of these mutations may reverse due to epistatic interactions with mutations elsewhere in the genome that arose and reversed the deleterious effect well before the deleterious mutation was fixed, and the beneficial combination of both mutations then rose together to fixation (Cowperthwaite et al. 2006). 3) A nonhomogeneous spatial distribution of alleles in the plant implies that deleterious alleles may persist longer in local subpopulations as long as they are not directly competing with better alleles present in their close neighborhood (Aguirre and Manrubia 2008). 4) If multiplicity of infection (MOI) is high, then cells can be coinfected by different variants and deleterious alleles may be easily complemented by shared common goods. Regarding this last explanation, experimental measures of MOI for TEV in tobacco rendered values <1.5 per cell (Tromas et al. 2014), although MOI has been estimated to be slightly higher (5–6 per cell) for soil-borne wheat mosaic potyvirus (Miyashita and Kishino 2010), in any case, MOI should be low enough to reduce the likelihood of complementation to explain the observed high frequency of some deleterious alleles. 5) Finally, a less interesting possible explanation is that these mutations are artifacts produced during the preparation and sequencing of the Illumina libraries. Given the inherent high error rate per site of this sequencing technique, in the 0.3–1% range. In the worse scenario, assuming a 1% error rate, then all the mutants discussed in this study would be sequencing errors. However, this possibility makes little sense in the light that some of the mutants had appeared in independent experiments. Even accepting that some of the mutants analyzed in this study were the result of technical errors, by characterizing them at the structural and phenotypic level we have provided relevant information on the selective and functional constraints operating on potyvirus’ 6K2.

### A Consideration on Epistasis and the Ruggedness of Adaptive Landscapes

Another interesting evolutionary aspect is the observation that the effect of some mutations is contingent to the presence of other mutations in the protein. For instance, mutation I32S in the 6K2·WT background shows completely different structural and phenotypic effects than when it appears in combination with mutation G33D (fig. 6). The same situation is true for mutation D44E in WT background and in presence of mutation F46L (fig. 6). In these two examples, fitness reversals from deleterious to beneficial effects result from epistatic interactions among mutations (Cowperthwaite et al. 2006). Epistasis determines the ruggedness of adaptive fitness landscapes and the accessibility of adaptive pathways (De Visser and Krug 2014). Epistasis has been shown to be pervasive in the genome of RNA viruses (reviewed in Elena et al. 2010), including TEV (Lalić and Elena 2012, 2015; Hillung et al. 2015), thus suggesting that TEV fitness landscape should be quite rugged in nature. Indeed, the ruggedness of TEV adaptive landscape has been shown to depend on the host species, being more rugged in a novel host (*Arabidopsis thaliana*) than in the natural one, *N. tabacum* (Cervera et al. 2016a).

Recent empirical studies exploring the accessibility of adaptive peaks for TEV in *Arabidopsisthaliana* at increasing mutational distances from the local optima have shown that the chances to return to this local peak decrease with mutational distance from it. At distances longer than one mutational step, viral populations tend to jump on the landscape and reach new distant peaks (Cervera et al. 2016b). When the contribution of adaptation, chance and contingency to evolution were evaluated, it turned out that adaptation, by large, was the most relevant factor, with contingency and chance event playing similar roles (Cervera et al. 2016b).

## Conclusions

Our results allow us to hypothesize a possible model of 6K2 molecular evolution in which different domains of the protein are subjected to different selective pressures: although the N-terminal domain is evolving under purifying selection, the transmembrane domains are more evolvable but with most mutations either retaining the α-helices or fusing them into a longer one, and the disordered C-terminal domain being also able to accommodate mutations. Mutations that are predicted in silico to have a major impact in 6K2 function result in asymptomatic or (most likely) no infections, whereas mutations predicted to have weaker functional effects result in infections which are similar to those induced by the WT virus. The in vivo estimated fitness effects of mutations are negatively associated with the ability to induce symptomatic infections and to the functional effects estimated in silico, which suggests that they strongly depend on the composition of the viral quasispecies. Observations also suggest a model in which a negative tradeoff may exist between within-host replicative fitness and severity of symptoms, with beneficial mutations being those associated with weaker symptoms and slower disease progression. This tantalizing possibility needs future experiments to further explore the evolution of this essential tiny viral protein.

## Supplementary Material

Supplementary data are available at *Genome Biology and Evolution* online.

## Supplementary Material

Supplementary Data
