# Supplementary material for: Mutagenesis Scanning Uncovers Evolutionary Constraints on Tobacco Etch Potyvirus Membrane-Associated 6K2 Protein
Source: Genome Biol Evol. 2019 Mar 27;11(4):1207–22. doi: 10.1093/gbe/evz069 (PMC6482416; doi:10.1093/gbe/evz069)
Supplement: Supplementary Data [file evz069_supp.zip › Supplementary Material.docx]

| **Supplementary table 1**. Pairs of primers used for generating the mutant 6K2 sequences. | |
| --- | --- |
| I32S | |
| Primer F | 5' GTCTGTGTTAAGTGGTGGTGGATGGATGC 3' |
| Primer R | 5' GCATCCATCCACCACCACTTAACACAGAC 3' |
| I32S-G33D | |
| Primer F | 5' GTCTGTGTTAAGTGATGGTGGATGGATGC 3' |
| Primer R | 5' GCATCCATCCACCATCACTTAACACAGAC 3' |
| G34D | |
| Primer F | 5' GTGTTAATTGGTGATGGATGGATGCTTG 3' |
| Primer R | 5' CAAGCATCCATCCATCACCAATTAACAC 3' |
| G34V | |
| Primer F | 5' GTGTTAATTGGTGTTGGATGGATGCTTG 3' |
| Primer R | 5' CAAGCATCCATCCAACACCAATTAACAC 3' |
| G34R/G35A | |
| Primer F | 5' GTTAATTGGTCGTGCATGGATGCTTGCAAC 3' |
| Primer R | 5' GTTGCAAGCATCCATGCACGACCAATTAAC 3' |
| G34S/A39S (primers to insert the G34S mutation) | |
| Primer F | 5' GTTAATTGGTAGTGGATGGATGCTTGC 3' |
| Primer R | 5' GCAAGCATCCATCCACTACCAATTAAC 3' |
| G34S/A39S (primers to insert the A39S mutation) | |
| Primer F | 5' GGATGGATGCTTTCAACGTACTTCAAG 3' |
| Primer R | 5' CTTGAAGTACGTTGAAAGCATCCATCC 3' |
| G35V | |
| Primer F | 5' GTTAATTGGTGGTGTATGGATGCTTGC 3' |
| Primer R | 5' GCAAGCATCCATACACCACCAATTAAC 3' |
| A39V | |
| Primer F | 5' GGATGGATGCTTGTAACGTACTTCAAG 3' |
| Primer R | 5' CTTGAAGTACGTTACAAGCATCCATCC 3' |
| A39E | |
| Primer F | 5' GGATGGATGCTTGAAACGTACTTCAAG 3' |
| Primer R | 5' CTTGAAGTACGTTTCAAGCATCCATCC 3' |
| D44E | |
| Primer F | 5' CGTACTTCAAGGAAAAGTTCAATGAAC 3' |
| Primer R | 5' GTTCATTGAACTTTTCCTTGAAGTACG 3' |
| D44E/F46L | |
| Primer F | 5' CGTACTTCAAGGAAAAGTTAAATGAAC 3' |
| Primer R | 5' GTTCATTTAACTTTTCCTTGAAGTACG 3' |

**Supplementary fig. 1.** Strategy for cloning and sequencing the “doped” 6K2 library into the infectious clone pMTEV by replacing the WT version of 6K2 by the mutant library. (A) Cloning process of the mutated amplicons into the linearized pMTEV plasmid. (B) Distribution of number of mutations per plasmid transformant. (C) Sequencing library preparation strategy.


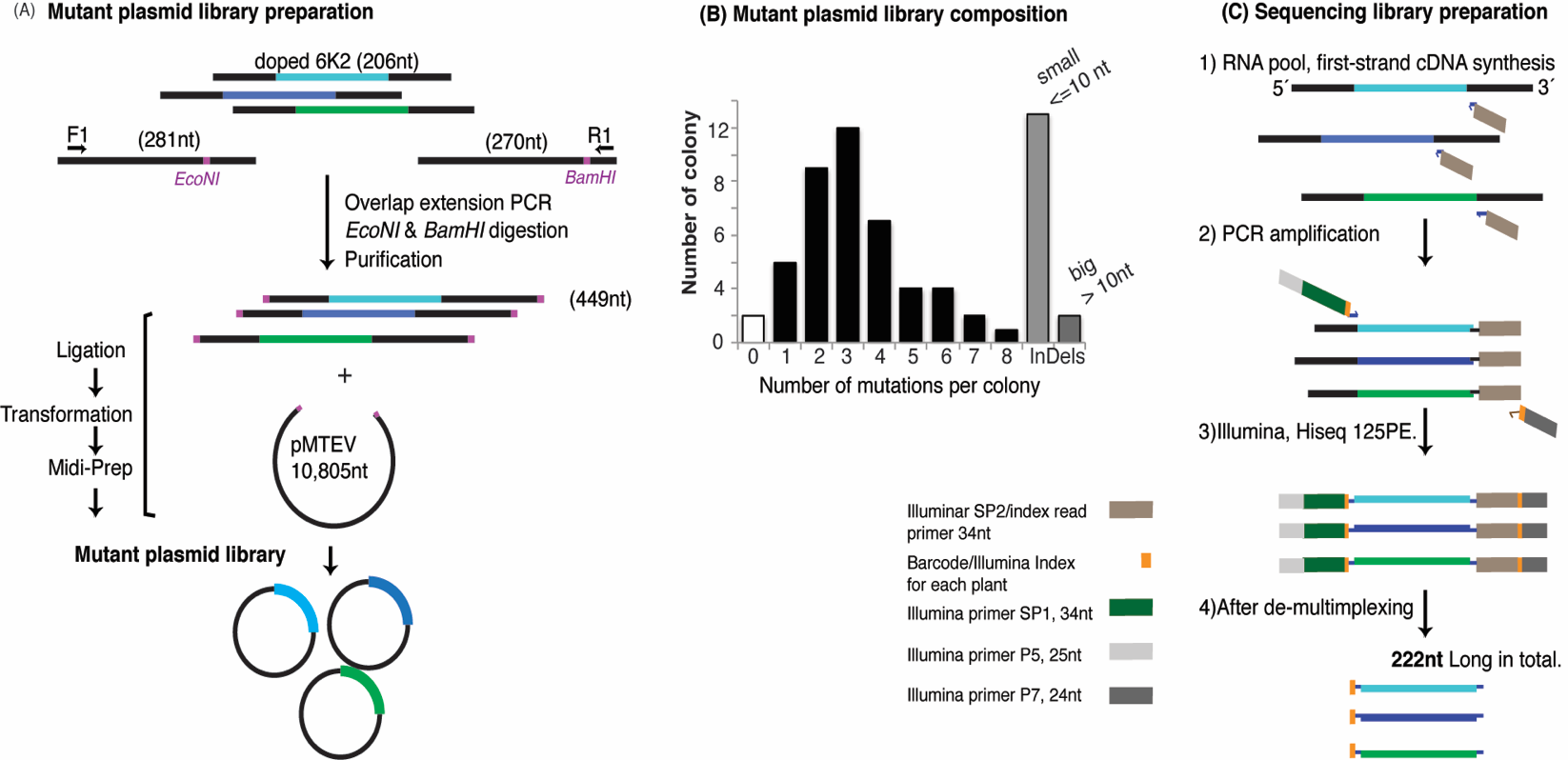


**Supplementary fig. 2.** Representative symptoms induced by TEV WT and mutant clones carrying the different 6K2 mutants. Plants were inoculated with equal amounts of RNA transcribed *in vitro* (see Material and Methods section). Pictures were taken 8 dpi for all the variants except for I32S/G33D and G34R/G35A that were taken 17 dpi.

**Supplementary file 1**. Primers used for preparation of the variants library and confirmation of the clones (Excel format).
